# Clinical stage I synchronous bilateral testicular germ cell tumor with different histopathology: a case report

**DOI:** 10.11604/pamj.2020.37.319.26267

**Published:** 2020-12-07

**Authors:** Carlos Eduardo Salazar-Mejía, Blanca Otilia Wimer-Castillo, Gisela García-Arellano, Raquel Garza-Guajardo, Oscar Vidal-Gutiérrez, Omar Alejandro Zayas-Villanueva, Francisco Emilio Vera-Badillo

**Affiliations:** 1 Centro Universitario Contra el Cáncer, Hospital Universitario “Dr. Jose Eleuterio Gonzalez”, Universidad Autonoma de Nuevo Leon, Monterrey, Mexico,; 2 Internal Medicine Department, Facultad de Medicina, Hospital Universitario “Dr. Jose Eleuterio Gonzalez”, Universidad Autonoma de Nuevo Leon, Monterrey, Mexico,; 3 Department of Pathology and Cytopathology, Facultad de Medicina, Hospital Universitario “Dr. Jose Eleuterio Gonzalez”, Universidad Autonoma de Nuevo Leon, Monterrey, Mexico,; 4 Department of Oncology, Kingston General Hospital, Ontario, Canada

**Keywords:** Testicular cancer, bilateral, young men, case report

## Abstract

Bilateral testicular germ cell tumors (BTGCT) occur in 1 to 4% of patients with testicular cancer and of these, 10-15% are synchronous. Overall, BTGCT represents less than 0.5% of all new cases of testicular cancer. There are few reports in the literature of synchronous BTGCT with different histology. We present the case of a 30-year-old man who presented to our genitourinary tumor unit with a bilateral increase of testicular volume. After initial assessment, a testicular ultrasound showed the presence of solid tumors in both testes. Staging studies were negative for metastatic disease. The patient was referred to the fertility clinic for sperm banking and later underwent a bilateral radical orchiectomy. The histopathology evaluation revealed a 5.5 cm right-sided mixed germ cell tumor and a 1.5 cm left-sided testicular seminoma. Because patient's poor compliance for surveillance was identified as a risk factor for relapse and poor outcome, adjuvant chemotherapy was favored. The patient underwent one cycle of bleomycin, etoposide and cisplatin (BEP). After four years of follow up, the patient shows no evidence of relapse, either clinically or radiologically. In men unlikely to carry out successful surveillance; active treatment is the preferred option for preventing disease recurrence, even in patients with no risk factors. The physician must consider all available therapeutic measures in this scenario to achieve the best possible therapeutic result.

## Introduction

Testicular cancer is the most common solid malignancy affecting men between the ages 15 and 35 [1]. In the United States, it is estimated that in 2020 there will be 9,610 new cases of testicular cancer, which represent 0.5% of new cancer cases [2]. Bilateral testicular germ cell tumors (BTGCT) occur in 1 to 4% of patients with testicular cancer [3]. Of BTGCT, 80-85% present as metachronous tumors, while 10-15% are synchronous. This represents less than 0.5% of all cases of testicular cancer [4]. Here, we present a case report of a patient with BTGCT and a review of the literature attainable to this type of rare presentation.

## Patient and observation

**Figure 1:**
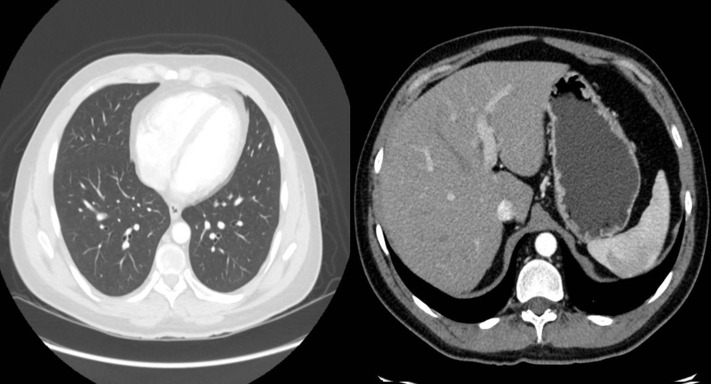
contrasted thoracoabdominal CT negative for lymph node involvement or metastatic disease

A 30-year-old man attended our office complaining of bilateral increase of testicular volume. After initial evaluation, a testicular ultrasound reported the presence of solid tumors in both testes. Pre-orchiectomy serum levels of β-hCG were 2.34 mIU/mL (normal range: <5.0 mIU/mL), AFP 17.9 ng/mL (normal range: <7.0 ng/mL), lactate dehydrogenase 584 IU/L (normal range: 91-180 IU/L). A CT scan of the chest, abdomen and pelvis did not show any evidence of lymph node involvement or metastatic disease (Figure 1).

**Figure 2:**
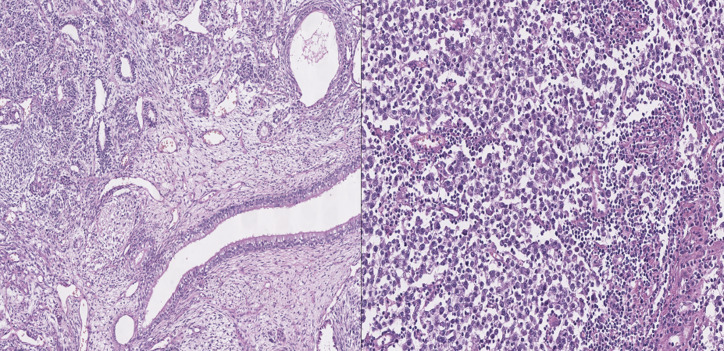
right testicular mixed germ cell tumor (left panel) associated with a left testicular seminoma (right panel)

The patient was referred to the fertility clinic for sperm banking and later underwent a bilateral radical orchiectomy. Histopathological assessment reported a 5.5 cm right-sided testicular mixed germ cell tumor (45% classic seminoma component, 40% mature and immature teratoma and 15% endodermic sinuses) and a 1.5 cm left-sided seminoma. Epididymis and rete testis were free of malignancy, lymphovascular invasion was absent and resection margins were free from tumor (Figure 2).

Tumor markers 2 weeks post-orchiectomy were as follows: β-hCG 1.2 mIU/mL, AFP 3.38 ng/mL, and lactate dehydrogenase 606 IU/L. Treatment options were discussed with the patient, including surveillance and adjuvant chemotherapy. Because patient´s poor compliance for surveillance was identified as a risk factor for relapse and poor outcome, adjuvant chemotherapy was favored. He underwent one cycle of bleomycin, etoposide and cisplatin (BEP). After four years of follow up, our patient shows no evidence of relapse, either clinically or radiologically.

## Discussion

In men with testicular cancer, a greater risk of developing testicular carcinoma in the contralateral testis has been described [5]. In the presence of a BTGCT, the absence of lymphatic or vascular connections between the two testes points towards two different primary tumors; it has also been postulated that the different histology originates from independent genetic alterations in each gonad [6].

Most cases of synchronous BTGCT present seminoma as a bilateral histological pattern, with only a few cases of BTGCT of different histological origin being reported in the literature to date [4,6,7]. BTGCT usually present in younger men (mean age at diagnosis 29 vs 34 years) with no significant difference in the initial clinical stage or in overall survival when compared to unilateral tumors [4]; however, the rarity of this presentation makes further analysis in this regard difficult. Table 1 summarizes synchronous BTGCT cases of different histology in men older than 18 years reported in English-language literature.

**Table 1:** reported cases of clinical stage I synchronic bilateral testicular germ cell tumors with a different histology

Author	Year	Country	Age at diagnosis, years	Histology (right/left testis)	Treatment received	Outcome, follow-up
Willis and Hajdu	1972	USA	22	ECA/seminoma	BO+RPLND+chemotherarpy	No evidence of disease (6 months)
Goodwin	1975	USA	27	MTI/seminoma	BO + RT	No evidence of disease (6 months) lung metastases (10 months)
Matsuhima	1987	Japan	30	Teratoma/seminoma	BO+ RPLND + RT	No evidence of disease (15 months)
Dieckmann	1988	Germany	32	Teratoma/seminoma	BO + RPLND	No evidence of disease (12 months)
Kratzik	1991	Austria	29	MTI/seminoma	BO+RPLND+chemotherapy	No evidence of disease (28 months)
Ondrus	1993	Slovakia	27	ECA/seminoma	BO	No evidence of disease (7 months)
Redmond	1995	USA	39	Seminona/ NSGCT	BO	Died of disease (24 months)
Tavolini	1999	Italty	22	NSGCT/seminoma	BO + RPLND	No evidence of disease (72 months)
Carrión-López	2008	Spain	37	Seminoma/mixed GCT	BO + chemotherapy	No evidence of disease (10 months)
Anastasiou	2015	Greece	40	ECA/seminoma	BO + chemptherapry	No evidence of disease (6 months)
Salazar-Mejía	2020	Mexico	30	Mixed GCT/seminoma	BO + chemotherapy	No evidence of disease (55 months)

BO: bilateral orchiectomy; RT: radiation therapy; GCT: germ cell tumor; ECA: embryonal carcinoma; RPLND: retroperitoneal lymph node dissection; MTI=malignant teratoma intermediate; NSGCT: nonseminomatous germ cell tumor; YST: yolk sac tumor; TSS: testis-sparing surgery *blank fields indicate no information available

Within the initial imaging approach of these tumors, the histological type could be correlated with its ultrasonographic appearance, since testicular seminomas are usually uniform and of regular borders with areas of necrosis in some cases, while embryonal carcinomas are irregular tumors that can show hemorrhagic areas [8]. Standard treatment for all testicular tumors in adults is radical inguinal orchiectomy; however, in selected patients with bilateral tumors, conservative procedures can be performed such as partial orchiectomy (tumor volume less than 30% of the testicular volume or smaller than 25mm confined to the testis and preoperative testosterone levels within normal range) or a testis-sparing surgery followed by CT in the setting of clinical stage (CS) I bilateral seminoma [5,7].

It is important to discuss the possibility of sperm banking before starting the proposed treatment, in order to preserve fertility. The need for indefinite androgenic replacement therapy and the psychological disorders associated with castration must also be evaluated. The pattern of subsequent management of BGCT is usually directed at the tumor with the highest stage and/or the worse histopathological characteristics. The prognosis of these patients is also based on this premise.

Available options for men with seminomatous germ cell tumors (SGCT) in CS I after surgery include surveillance, 1-2 cycles of carboplatin (CBP) AUC 7 or radiotherapy (20-30 Gy). In the largest retrospective series reported, 1954 Danish patients with CS I SGCT were monitored and followed for a period of 15 years; the cancer-specific survival (CSS) at 15 years was 99.3% [9]. The risk factors for recurrence are tumor size >4 cm, invasion of the rete testis and lymphovascular invasion [10]. In men without these risk factors who are under surveillance, recurrence rates of only 6% are described [11]. The risk of occult metastatic disease in CS I SGCT is around 12%, while in high-risk disease is 32% [12]. In a prospective study of the Spanish group, patients without risk factors or with only one risk factor (rete testis invasion or size >4 cm) were subjected to surveillance, while those who had both risk factors received CBP AUC 7 X 2 cycles. The DFS at 3 years was 88.1% for patients under surveillance and 98% for those treated with chemotherapy; however, this did not impact the 3-year overall survival [13]. In the non-inferiority study of Oliver *et al*. comparing both adjuvant options, radiotherapy (RT) and one cycle of CBP AUC 7, provided similar results in RFS at 5 years [14]. It is important to mention that up to 70% of patients who present recurrence during surveillance are treated with RT alone because these recurrences are usually low-volume retroperitoneal disease [12].

Regarding CS I non-seminomatous germ cell tumors (NSGCT), treatment options include surveillance, nerve-sparing retroperitoneal lymph node dissection (NS-RPLND) or BEP X 1 cycle [1]. The percentage of cure regardless of the treatment chosen ranges from 98 to 100% since relapses are usually successfully treated with BEPX3 or EPX4 [15]. Global recurrence rates at 5 years are close to 30% with an average time to recurrence of 5-7 months. The main risk factors for recurrence are the presence of lymphovascular invasion, a component of embryonal carcinoma >50% of the tumor specimen and invasion of the rete testis.

In the prospective study by Tandstad *et al*. recurrence rates of 13.2% were observed in tumors that did not present vascular invasion and that were monitored after orchiectomy. In patients who presented vascular invasion and who were only monitored, the recurrence rate was 41.7% [16]. A prospective study compared patients without vascular invasion and monitored versus those with vascular invasion and subjected to adjuvant BEP for 2 cycles, evidencing recurrence rates of 17.3% versus 1.1%, respectively. This risk-based strategy resulted in a 3-year overall survival of 99.1% in the total study population [17].

A phase II study evaluated the role of 1 cycle of adjuvant BEP in 40 patients with CS I NSGCT associated with vascular invasion and/or an embryonic component >50% in the orchiectomy specimen. After a follow-up of 15 years, only 1 patient had a relapse (2.5%), who died as a result of disease progression. In addition, 3 patients (7.5%) presented another primary neoplasm [18]. In the prospective study by Huddart *et al*. treatment with 1 cycle of adjuvant BEP in high-risk NSGCT resulted in a 2-year recurrence rate of 1.3%, with a 2-year OS of 99.2%; results similar to those previously described with 2 cycles of this scheme [19].

In a phase III superiority study carried out by Albers *et al*. 382 patients with CS I NSGCT treated with orchiectomy were randomized 1: 1 to receive 1 cycle of BEP or to NS-RPLND. Of the orchiectomy specimens in both groups, 41% had lymphovascular invasion. In the surgical group 18% of patients were re-categorized as CS II or higher after NS-RPLND and therefore, underwent BEP for 2 cycles. In the intention-to-treat population, after a mean follow-up of 4.7 years, 2 and 15 recurrences were reported in the chemotherapy group and the NS-RPLND group, respectively (p=0.0011). The absolute difference in RFS at 2 years was 7.59%. The clinical efficacy of only 1 cycle of BEP in preventing disease recurrence was corroborated, reaching a RFS close to 100% at 2 years [20]. However, due to the toxicity described, the patient who will undergo this intervention must be carefully chosen.

## Conclusion

Close surveillance has emerged as the preferred option in men with good adherence to follow-up and with access to economic resources to afford the necessary diagnostic tests; however, in patients with a low probability of carrying out successful surveillance, active treatment is an adequate and effective option to prevent disease recurrence, even in patients without risk factors.
